# Modification of the tumour microenvironment *via* exosomal shedding of sphingosine 1-phosphate receptor 2 by breast cancer cells

**DOI:** 10.18632/oncotarget.25793

**Published:** 2018-07-24

**Authors:** Stuart M. Pitson, Jason A. Powell

**Affiliations:** Stuart M. Pitson: Centre for Cancer Biology, University of South Australia and SA Pathology, North Tce, Adelaide SA, Australia

**Keywords:** sphingosine 1-phosphate, exosomes, breast cancer, tumour microenvironment

Sphingosine-1 phosphate (S1P), formed by the phosphorylation of sphingosine by the sphingosine kinases, is a bioactive signaling lipid that can promote cell proliferation, survival, differentiation and migration [1]. S1P has been widely implicated in cancer initiation, progression and chemotherapeutic resistance *via* its actions as a ligand for a family of five S1P-specific G protein-coupled receptors (GPCRs), named S1P_1_ to S1P_5_, and as an intracellular second messenger that can alter gene transcription [2]. S1P can engage its GPCRs in both an autocrine and paracrine fashion to promote classical GPCR signaling cascades and biological responses. Specifically S1P_2_ is located on the plasma membrane and coupled to G_i_, G_q_ and G_12/13_, and upon S1P binding activates phospholipase C, Rho and ERK1/2 signaling pathways. Despite S1P being classically involved in tumorigenic signaling, the role of S1P_2_ in this process has been ambiguous with different studies indicating that this receptor can either promote [3-5] or inhibit [6] tumorigenesis.

El Buri et al. [7] in this issue of Oncotarget have identified a new novel mechanism by which S1P_2_ regulates the tumour microenvironment. The authors hypothesize that, like the sphingosine kinases [8], the S1P receptors might also be shed from breast cancer cells in exosomes to regulate the tumour microenvironment. Initial experiments indeed showed that S1P_2_ was released into the conditioned medium from MDA-MB-231 breast cancer cells, which induced robust ERK1/2 activation and DNA synthesis/cell proliferation of mouse embryonic fibroblasts (MEFs). Using a combination of exosome isolation, western blotting, immunofluorescence and electron microscopy the authors clearly demonstrated the S1P_2_ was derived from exosomes shed from breast cancer cells and taken up by the MEFs. To exclude the possibility that additional cargo within the exosomes contribute to the activation of ERK-1/2 signaling cascade in fibroblasts, the authors knocked down S1P_2_ in MDA-MB-231 cells and showed exosomes from scrambled but not S1P_2_ siRNA treated MDA-MB-231 cells promoted ERK-1/2 activation. Unexpectedly, however, the ERK1/2 activation by this conditioned medium was insensitive to the S1P_2_ receptor antagonist JTE-013, implying that the S1P_2_ released from breast cancer cell-derived exosomes is no longer able to bind JTE-013. Indeed, the evidence suggested that this exosomal S1P_2_ is taken up by fibroblasts and processed at the N-terminus to a smaller (36 kDa versue the normal 40 kDa protein) constitutively active form. This N-terminal truncated form of S1P_2_ induced significant morphological rounding of MEFs providing further evidence that this form of S1P_2_ is biologically active. Interestingly, the authors also identified a similarly truncated species of S1P_2_ in the MEFs. The appearance of this endogenous MEF-derived truncated S1P_2_, however, was blocked by JTE-013 suggesting that, unlike the exosomal-derived S1P_2_, it may be processed in response to engagement of S1P.

The identification of the putative protease present in MEFs that cleaves the N-terminus of exosomal S1P_2_ will be of great interest to the field. To this end El Buri et al. [7] identified a putative intramembrane metalloprotease cleavage site near the N-terminus of S1P_2_. While it remains to be determined if this is the physiologically relevant cleavage site, notably, a S1P_2_ mutant truncated to this residue phenocopied the exosomal-derived truncated S1P_2_ in activating ERK-1/2 signaling. El Buri et al. have also begun to address the mechanism by which a truncated S1P_2_ induces ligand independent activation of ERK-1/2 signaling. Using the crystal structures of the homologous S1P_1_ receptor they generated a structural model of S1P_2_. From this they hypothesized that in the inactive state the N-terminus of S1P_2_ may tension its transmembrane helix (TM)1 to maintain a compressive force on TM7. This potentially results in stabilization of the interface between the intracellular ends of TM7 and TM6 constraining S1P_2_ signaling. Removal of the N-terminal peptide is predicted to relax TM1 resulting in separation of TM6 and TM7 permitting G protein engagement and constitutive activity of the receptor.

These studies raise a number of critical questions that should be addressed in the future. Firstly, what are the consequences of exosomal S1P_2_ for the tumor microenvironment *in vivo*, and does it contribute to a microenvironment conducive to metastatic spread? Secondly, what regulates the release of exosomal S1P_2_ from breast cancer cells, and does this occur in other cancers? Thirdly, what is the exact cleavage site and the accompanying protease that processes exosomal S1P_2_. Answers to these questions may uncover novel therapeutic targets to manipulate the supportive tumour microenvironment.

These findings of El Buri et al. [7] are important as this is the first demonstration of exosomal-derived S1P_2_ which has the potential to regulate the tumour microenvironment. In addition to breast cancer, this may have broad reaching implications to other cancers that have a dependence on S1P_2_ and provide novel avenues for therapeutic development.
